# Ammonium [^11^C]thiocyanate: revised preparation and reactivity studies of a versatile nucleophile for carbon-11 radiolabelling†
†Electronic supplementary information (ESI) available: Experimental details and HPLC traces. See DOI: 10.1039/c7md00425g


**DOI:** 10.1039/c7md00425g

**Published:** 2018-07-02

**Authors:** Tom Haywood, Sara Cesarec, Steven Kealey, Christophe Plisson, Philip W. Miller

**Affiliations:** a Department of Chemistry , Imperial College London , South Kensington , SW7 2AZ , London , UK . Email: philip.miller@imperial.ac.uk ; Tel: +44 (0)2875942847; b Imanova Limited , Imperial College London , Hammersmith Hospital , Burlington Danes Building, Du Cane Road , London , W12 0NN , UK

## Abstract

Herein we report the preparation of ammonium [^11^C]thiocyanate *via* the reaction of [^11^C]CS_2_ with ammonia. The [^11^C]SCN^–^ ion is demonstrated as a potent nucleophile that can be used to readily generate a range of ^11^C-labelled thiocyanate molecules in high conversions. Furthermore, novel ^11^C-labelled thiazolone molecules can be easily prepared from the intermediate α-thiocyanatophenones *via* an acid mediated cyclisation reaction.

## Abstract 2

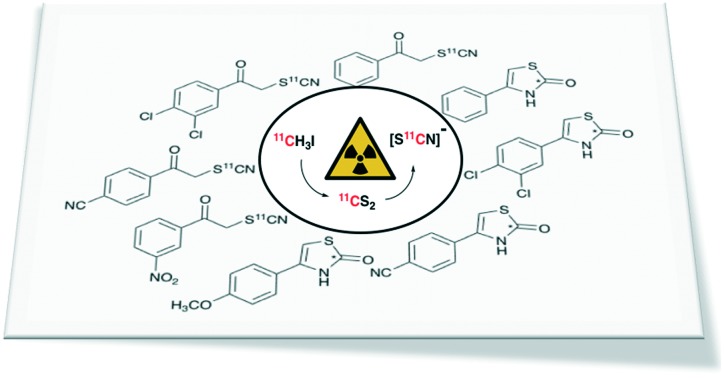
Ammonium [^11^C]thiocyanate, produced from [^11^C]CS_2_, was used to efficiently radiolabel a range of C-11 thiocyanate and thiazolone molecules.

## 

Positron emission tomography (PET) is now a widely used clinical and research imaging technique for the study and diagnosis of a range of neurological, oncological and cardiac conditions.^1^–^5^ The expansion of PET imaging centres worldwide over the past decade has led to an accompanying increase in demand for both new and existing PET tracers. The development of PET tracers presents a number of key chemical and biological challenges due to the short half-lives of positron-emitting nuclides, the peculiarity of tracer level chemical reactions, the complexity of biological interactions, and the strict working environment required for safe handling of radioactive substances. The synthetic chemistry hurdles are particularly apparent considering that the half-lives of the two most common PET nuclides, carbon-11 and fluorine-18, are only 20.4 min and 109.7 minutes, respectively. For example, a clinical radiopharmaceutical carbon-11 tracer production involving radiosynthesis, purification and formulation, typically needs to be complete within a 30–45 min timeframe. The development of new tracers is further confounded by the limited number of available PET precursors and methods for labelling, meaning that there are only a certain number of reactions that can be used to introduce the radionuclide.^6^ This limitation clearly restricts access to what can ultimately be radiolabelled and where on the target molecule the radionuclide can be introduced.

Despite these challenges, an array of novel fluorine-18 and carbon-11 labelling methods have been reported in recent years to try overcome these challenges.^7^–^12^ Carbon-11 is typically incorporated into tracers *via* well-established methylation protocols using [^11^C]CH_3_I or the more reactive [^11^C]CH_3_SO_3_CF_3_.^6^,^13^ Carbon-11 methylation is, however, unsuitable for labelling many interesting biological molecules, either due to the absence of a methyl group on the target molecule or due to unfavourable metabolism of the labelled methyl group *in vivo*. A range of other reactive small molecule carbon-11 reagents have therefore been developed to label in different positions. For example [^11^C]CO_2_,^14^ [^11^C]CO,^15^ [^11^C]HCN,^11^ [^11^C]CH_2_O^16^ and [^11^C]COCl_2_ 17 have been used to radiolabel carbonyl and cyano functional groups. Recently, our group reported the production of a novel carbon-11 labelling reagent, [^11^C]carbon disulfide, and described its initial reactivity studies for the efficient labelling of organosulfur compounds such as dithiocarbamates, thioureas and other biological molecules.^18^–^20^ Herein, we report the facile production of ammonium [^11^C]thiocyanate, prepared *via* the reaction of [^11^C]CS_2_ with ammonia, and describe preliminary radiolabelling reactions of this versatile nucleophilic reagent.

The thiocyanate ion is a ‘pseudohalide’ and potent nucleophile that can react either *via* the nitrogen atom or sulfur atom. It has been widely use to prepare organic thiocyanates and a range of other sulfur containing compounds (Scheme 1).^21^ The thiocyanate ion is attractive from a radiolabelling perspective because of its reactivity and potential to form a wide range of organosulfur derivatives. [^11^C]Thiocyanate has been previously reported, however, its synthesis relies on the production of [^11^C]HCN, which is not readily accessible in most PET centres, thus limiting its potential wider use. Typically, [^11^C]SCN^–^ is prepared by first producing [^11^C]HCN, followed by conversion to a salt and then reaction with a sulfur source. The first reported method describes a two-step process where [^11^C]HCN is passed through a phosphorous pentoxide trap into a solution of sodium or potassium hydroxide to generate [^11^C]CN^–^, which is then reacted with elemental sulfur to give [^11^C]SCN^–^ (Scheme 2, top).^22^,^23^ In an alternative approach, [^11^C]HCN can be converted to [^11^C]NaSCN *via* bromination in triglyme to give [^11^C]BrCN followed by distillation over antimony and reaction with sodium sulfide (Scheme 2, top).^24^ In an effort to prepare a range of sulfur based labelled compounds we sought to explore alternative methods to synthesise [^11^C]SCN^–^ that circumvent the need for specialised [^11^C]HCN synthesis equipment (Scheme 2).

**Scheme 1:**
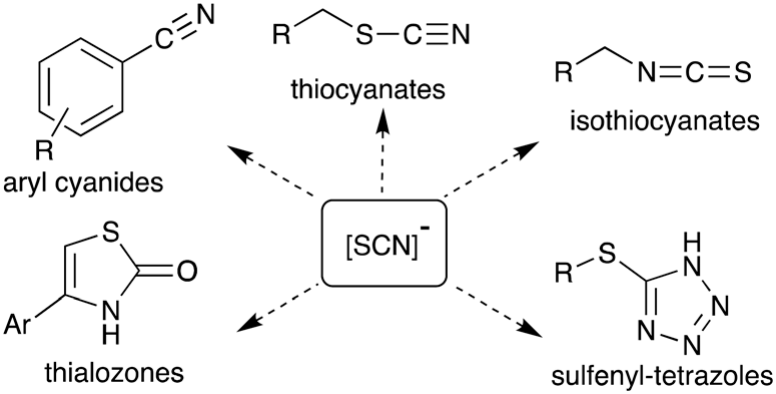
A selection of organic molecules that can be prepared using the thiocyanate ion.

**Scheme 2:**
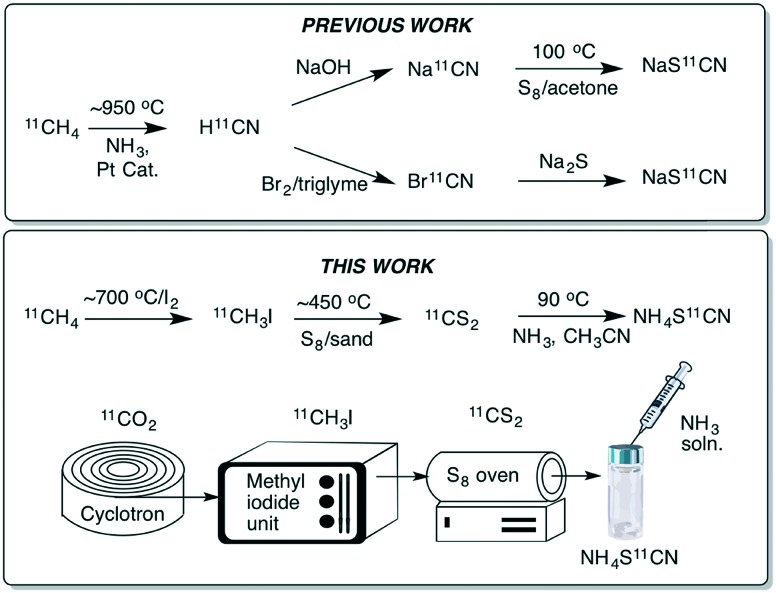
Previously reported routes to [^11^C]SCN^–^ relying on generating [^11^C]HCN first (top); this work, which reports the synthesis of [^11^C]SCN^–^*via* [^11^C]CS_2_ (bottom).

Our recent work on the development of [^11^C]CS_2_ led us to investigate this reagent as an alternative approach to prepare [^11^C]SCN^–^. Carbon disulfide is known to react with ammonia to give ammonium dithiocarbamate. On heating ammonium dithiocarbamate to 90 °C, ammonium thiocyanate is generated along with one equivalent of H_2_S. We previously reported a fast and easily accessible route to [^11^C]CS_2_*via* the reaction of sulfur vapour with [^11^C]CH_3_I. Unlike [^11^C]HCN, [^11^C]CH_3_I is a much more commonly used carbon-11 labelling reagent that is prevalent in many PET centres. In our initial experiments to prepare [^11^C]SCN^–^, a stream of gaseous [^11^C]CS_2_ was passed into an ammonia solution (0.1 M in MeCN) and heated to 90 °C for 5 min. Analysis of the crude reaction mixture confirmed [^11^C]NH_4_SCN formation, albeit in low radiochemical yields (RCY) (<10%, Table 1). We hypothesised that passing the [^11^C]CS_2_ gas stream directly through the ammonia solution resulted in significant loss of the volatile ammonia, hence greatly reducing its concentration and therefore limiting the reaction. To circumvent this, the order of addition was changed to avoid ammonia loss; first trapping the [^11^C]CS_2_ in acetonitrile (1 mL), then adding the ammonia solution (50 μL, 2 M NH_3_ in CH_3_OH) and heating to 90 °C for 5 min. Pleasingly, this resulted in near quantitative radiochemical conversion of [^11^C]CS_2_ to [^11^C]NH_4_SCN as observed by radio-HPLC (Table 1).

**Table 1:** Conversion of [^11^C]CS_2_ to [^11^C]NH_4_SCN *via* reaction with ammonia solution

^11^CS_2_ + 2NH_3_ → NH_4_S^11^CN + H_2_S		
Synthesis method^*a*^	RCY^*b*^ [^11^C]CS_2_ (%)	RCY^*b*^ [^11^C]NH_4_SCN (%)
A	90	10
B	1	99

^*a*^Method A: [^11^C]CS_2_ gas stream bubbled through ammonia solution (50 μL, 2 M NH_3_ in CH_3_OH, acetonitrile 1 mL) followed by heating to 90 °C for 5 min. Method B: addition of ammonia solution (50 μL, 2 M NH_3_ in CH_3_OH) to [^11^C]CS_2_ in acetonitrile (1 mL) followed by heating to 90 °C for 5 min.

^*b*^Non-isolated radiochemical yield (RCY) determined by analytical radio HPLC of the crude product based on conversion from [^11^C]CH_3_I. Average of minimum *n* = 3.

Given its ease of synthesis and potential utility as a nucleophile, we aimed to explore the reactivity of the [^11^C]thiocyanate ion for substitution reactions. A simple model reaction of [^11^C]NH_4_SCN with benzyl bromide was initially investigated. In a typical labelling experiment, a solution of benzyl bromide was injected into the reaction vial containing *in situ* generated [^11^C]NH_4_SCN, and the mixture heated for 5 min at 90 °C (Scheme 3).

**Scheme 3:**
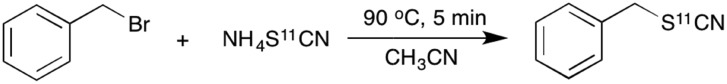
Substitution reaction of benzyl bromide with [^11^C]NH_4_SCN to generate benzyl [^11^C]thiocyanate.

The crude product mixture was analysed by radio-HPLC and the product identity confirmed by comparison of the radioactive sample with unlabelled reference material. [^11^C]NH_4_SCN was found to react cleanly and quickly with benzyl bromide showing virtually quantitative conversion to benzyl [^11^C]thiocyanate (Chart 1). Encouraged by this result, we proceeded to explore this reaction with a range of α-ketobromides to generate their respective ^11^C-labelled α-thiocyanatophenones. We discovered that excellent conversions could be achieved for the C-11 thiocyanation of all seven α-ketobromides we tested, within a 5 min reaction time (Chart 1). Lower conversions were observed for the reaction of the diphenyl substrate (Chart 1, entry 8), most likely due to the greater steric hindrance of this substrate inhibiting nucleophilic attack of the thiocyanate ion. [^11^C]NH_4_SCN was also found to react cleanly with mannose triflate, the precursor used for 2-([^18^F]fluoro)-2-deoxy-d-glucose synthesis, to give the labelled sugar thiocyanate derivative (**9**).

**Chart 1:**
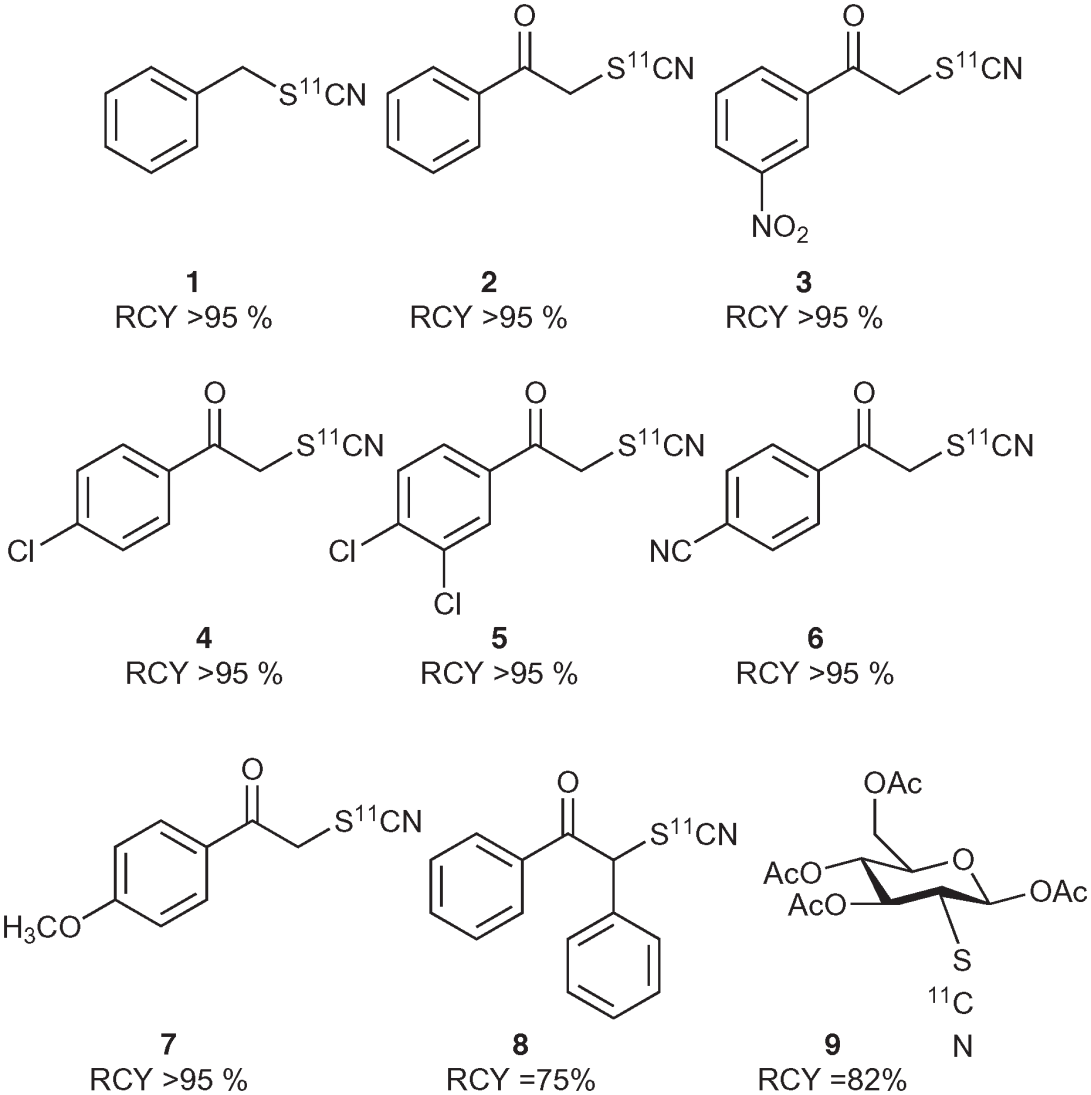
Radiolabelled thiocyanate molecules. Average of minimum *n* = 2.

Thiocyanate compounds are interesting intermediates because they can be converted into other organosulfur and heterocyclic structures.^25^–^27^ Thiazole structures are prevalent in a number of important naturally occurring compounds, for example thiamine and penicillin, and in a wide range of bioactive small molecules such as benzothiazoles, while the related thiazolones have been investigated for anti-cancer activity.^28^ Carbon-11 radiolabelling within the ring of such sulfur based heterocyclic structures is challenging, and to the best of our knowledge has not been achieved until now. Since thiazolones can be accessed in high yield from α-thiocyanatophenones *via* an acid mediated cyclisation reaction (Scheme 4), we sought to explore this pathway using our *in situ* generated α-[^11^C]thiocyanatophenones.^29^

**Scheme 4:**
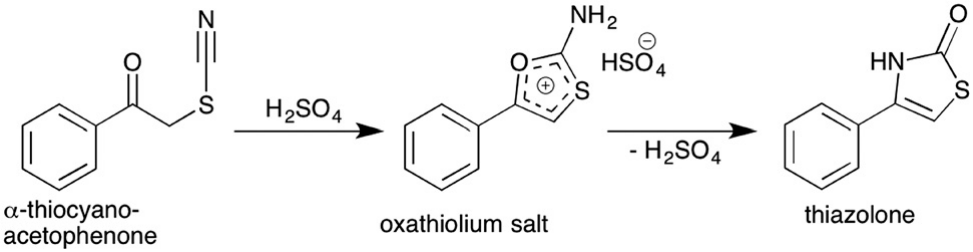
Acid mediated cyclisation of α-thiocyanatophenone which is hypothesised to proceed *via* the intermediate oxathiolium salt prior to thiazolone formation and elimination of sulfuric acid.

To test this in the radiochemistry laboratory, a solution of 1-phenyl-2-[^11^C]thiocyanatoethanone (**2**) was added to a mixture of sulfuric and acetic acid, and heated to 90 °C for 5 min. This resulted in complete conversion of **2** to the cyclised thiazolone, 4-phenylthiazol-2-[^11^C]one (**10**) (Scheme 5, Chart 2). This cyclisation reaction was then performed with the remaining phenyl-substituted α-[^11^C]thiocyanatophenones, producing the corresponding 4-phenylthiazol-2-[^11^C]ones in high radiochemical purity (70–95%, Chart 2).

**Scheme 5:**
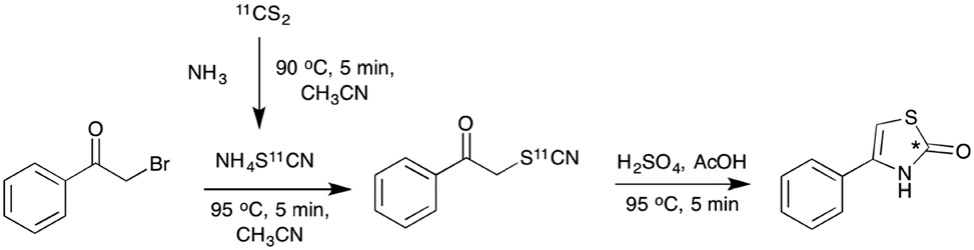
One pot radiolabelling of 4-phenylthiazol-2-[^11^C]one (**10**) *via* acid mediated cyclisation of the intermediate 1-phenyl-2-[^11^C]thiocyanatoethanone (**2**), (*) denotes the ^11^C labelling position.

**Chart 2:**
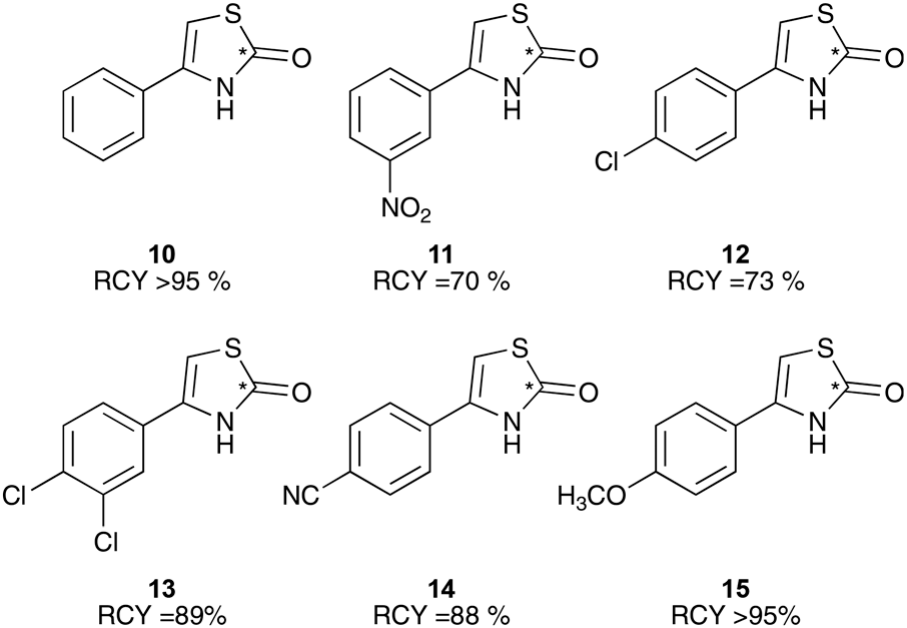
^11^C-Radiolabelled thiazolone molecules. Average of minimum *n* = 2.

In conclusion, we have developed a new synthetic route to ammonium [^11^C]thiocyanate *via* the reaction of ammonia with [^11^C]carbon disulfide. The speed and efficiency of this synthesis has enabled us to exploit the [^11^C]thiocyanate ion as a potent nucleophilic species for the preparation of a range of radiolabelled thiocyanate compounds in high radiochemical yields. Furthermore, we have been able to rapidly conduct cyclisation reactions with these α-[^11^C]thiocyanatophenones to generate unprecedented ^11^C-thiazolone molecules. We anticipate that this labelling route will find further applications for the preparation of carbon-11 based organosulfur tracers. Given that [^11^C]CS_2_ can be easily prepared from the widely available carbon-11 precursor [^11^C]CH_3_I we also expect that this method could be easily adopted by other PET centres with carbon-11 facilities.

We are grateful for financial support from the EPSRC (grant no. EP/L025140/1) and the Royal Society (grant no. RG110449).

## Conflicts of interest

The authors declare no competing interest.

## Supplementary Material

Supplementary information
