# Supplementary material for: Ammonium [11C]thiocyanate: revised preparation and reactivity studies of a versatile nucleophile for carbon-11 radiolabelling
Source: Medchemcomm. 2018 Jul 2;9(8):1311–4. doi: 10.1039/c7md00425g (PMC6096773; doi:10.1039/c7md00425g)
Supplement: Supplementary file 1 [file MD-009-C7MD00425G-s001.pdf]

Electronic Supplementary Material (ESI) for MedChemComm.
This journal is © The Royal Society of Chemistry 2018

Supporting information

Ammonium [^{11}C]thiocyanate: revised preparation and reactivity studies of a versatile nucleophile for carbon-11 radiolabelling

Tom Haywood,[a] Sara Cesarec,[a] Steven Kealey,[a] Christophe Plisson[b] and Philip W. Miller*[a]

[a]Department of Chemistry, Imperial College London, South Kensington, London, SW7 2AZ (UK), E-mail: philip.miller@imperial.ac.uk; Tel:+44(0)2875942847

[b]Imanova Limited, Burlington Danes Building, Imperial College London, Hammersmith Hospital, Du Cane Road, London, W12 0NN (UK)

General considerations: preparation of reference materials

Starting materials were of reagent grade and purchased from either Sigma-Aldrich or VWR International and used without further purification. ^{1}H and $^{13}C\{^{1}H\}$ NMR spectra were recorded on Bruker AV-400, AV-500, or DRX-400 spectrometers at 294 K unless otherwise stated. Chemical shifts are reported in ppm using the residual proton impurities in the solvents for ^{1}H NMR spectroscopy and the solvent for $^{13}C\{^{1}H\}$ NMR spectroscopy.

Ammonium thiocyanate

Carbon disulfide (0.6 mL, 10 mmol) was added to a solution of methanolic ammonia (2 M, 2 mL, 4 mmol) in acetonitrile (50 mL), and brought to reflux for 30 minutes. The solvent was then removed under reduced pressure to give a white precipitate in quantitative yield. ^{1}H NMR (400 MHz, CD_3CN) δ: 6.23. $^{13}C\{^{1}H\}$ NMR (101 MHz, CD_3CN) δ 117.3.

General thiocyanation procedure

Phenacyl thiocyanate compounds were prepared via a modified procedure to that reported by Zhao et al.[1] To a solution of bromoacetophenone precursor (5 mmol) in acetonitrile (15 mL) was added ammonium thiocyanate (0.38 g, 5 mmol) and brought to reflux for 1 hour. Typically, an off-white precipitate formed during the course of the reaction. The reaction mixtures were concentrated *in vacuo* and the precipitates were isolated in high yields via filtration and used without further purification. ^{1}H and $^{13}C\{^{1}H\}$ NMR spectroscopic data of prepared compounds are given below and conformed to those previously published in the literature.[1]

1-phenyl-2-thiocyanatoethanone (2)

^{1}H NMR (400 MHz, d_6-DMSO) δ: 8.09-7.95 (m, 2H), 7.81-7.68 (m, 1H), 7.59 (t, J = 7.7 Hz, 2H), 5.13 (s, 2H). $^{13}C\{^{1}H\}$ NMR (101 MHz, d_6-DMSO) δ: 192.8, 134.8, 129.4, 129.0, 113.3, 42.3.

1-(3-nitrophenyl)-2-thiocyanatoethanone (3)

^{1}H NMR (400 MHz, CD_3CN) δ: 8.75 (t, J = 2.0 Hz, 1H), 8.51 (ddd, J = 8.2, 2.2, 1.0 Hz, 1H), 8.36 (ddd, J = = 8.2, 2.2, 1.0, 1H), 7.83 (t, J = 8.0 Hz, 1H), 4.88 (s, 2H). $^{13}C\{^{1}H\}$ (101 MHz, CD_3CN) δ: 190.6, 135.7, 134.3, 130.6, 128.4, 123.1, 117.3, 111.7, 42.0.

1-(4-chlorophenyl)-2-thiocyanatoethanone (4)

^{1}H NMR (400 MHz, d_6-DMSO) δ: 8.10-7.97 (m, 2H), 7.73-7.60 (m, 2H), 5.09 (s, 2H). $^{13}C\{^{1}H\}$ NMR (101 MHz, DMSO-d_6) δ: 191.9, 139.7, 133.5, 130.9, 129.5, 113.1, 42.0.

1-(3,4-dichlorophenyl)-2-thiocyanatoethanone (5)

^{1}H NMR (400 MHz, $CDCl_3$) δ: 8.05 (d, *J* = 2.0 Hz, 1H), 7.79 (dd, *J* = 8.4, 2.1 Hz, 1H), 7.65 (d, *J* = 8.4 Hz, 1H), 4.69 (s, 2H). ^{13}C{^{1}H} NMR (101 MHz, $CDCl_3$) δ: 188.83, 139.71, 134.13, 133.45, 131.35, 130.37, 127.36, 111.22, 42.29.

1-(4-benzonitrile)-2-thiocyanatoethanone (6)

^{1}H NMR (400 MHz, CD_3CN) δ: 8.17-8.06 (m, 1H), 7.97-7.89 (m, 1H), 4.84 (s, 1H). ^{13}C{^{1}H} NMR (101 MHz, 101 MHz, CD_3CN) δ 191.3, 137.5, 132.9, 128.9, 117.3, 111.7, 42.2.

1-(4-methoxyphenyl)-2-thiocyanatoethanone (7)

^{1}H NMR (400 MHz, $CDCl_3$) δ: 8.00-7.89 (m, 2H), 7.07-6.94 (m, 2H), 4.74 (s, 2H), 3.92 (s, 3H). ^{13}C{^{1}H} NMR (101 MHz, $CDCl_3$) δ: 189.1, 164.8, 130.9, 126.9, 114.3, 112.1, 55.7, 42.9.

1,2-diphenyl-2-thiocyanatoethanone (8)

^{1}H NMR (400 MHz, $CDCl_3$) δ: 7.91-7.85 (m, 2H), 7.58-7.51 (m, 1H), 7.46-7.34 (m, 7H), 6.19 (s, 1H). ^{13}C{^{1}H} NMR (101 MHz, $CDCl_3$) δ: 192.3, 134.6, 134.5, 133.5, 130.0, 129.9, 129.5, 129.1, 128.5, 112.4, 62.7.

1,3,4,6-tetra-*O*-acetyl-2-thiocyanato-D-mannopyranose (9)

Mannose triflate (52 mg, 1 eqv) was dissolved in minimum amount of acetonitrile, followed by addition of ammonium thiocyanate (9 mg, 1.1 eqv) and stirred at 85°C for 24 hours. The crude reaction mixture was purified by preparative-LCMS to yield a white solid (20 mg, 48%). ^{1}H NMR (400 MHz, CD_3CN) δ: 5.97 (d, *J* = 9.0 Hz, 1H), 5.36 (dd, *J* = 11.1, 9.1 Hz, 1H), 5.15 – 5.05 (m, 1H), 4.31 – 4.21 (m, 1H), 4.10–4.06 (m, 1H), 4.06–4.02 (m, 1H), 3.53 (dd, *J* = 11.1, 9.1 Hz, 1H), 2.19 (s, 3H, CH_3CO), 2.10 (s, 3H, CH_3CO), 2.03 (s, 3H, CH_3CO), 2.02 (s, 3H, CH_3CO). ^{13}C{^{1}H} NMR (101 MHz, CD_3CN) δ: 170.2, 169.8, 169.4, 168.6, 117.3, 91.9, 72.3, 70.7, 68.5, 61.3, 50.3, 19.9, 19.8, 19.7. MS (ES+) m/z calculated for $C_{15}H_{19}NO_9S$: 389, found 412 $[M\text{-}Na]^+$ (100). Analytical LCMS trace elution time at 13.07 minutes, XBridge^{TM} C18 5µm (4.6 x100 mm) column with a gradient of methanol and water at 1 ml/min. Preparative LCMS elution time at 8.01 minutes, XBridge^{TM} Prep C18 5µm OBD^{TM} (19x100 mm) column with a gradient of methanol and water at 5 ml/min.

General thiazolone synthesis

Thiazolone compounds were prepared via a procedure to that reported by Pihlaja et al.[2] Briefly, phenacyl thiocyanate precursor (2.5 mmol) was added to acetic acid (20 mL) and heated until the

thiocyanate completely dissolved. Sulfuric acid (50 % v/v, 4 mL) was added and the solution brought to reflux for 20 minutes. The reaction mixture was then cooled to ambient temperature and poured into cold water which resulted in precipitation. The precipitates were isolated by filtration in high yields and washed with hexane. ^{1}H and ^{13}C{^{1}H} NMR spectroscopic data of prepared compounds are given below and conformed to those previously published in the literature.[2]

4-phenylthiazol-2-one (10)

^{1}H NMR (400 MHz, d_6-DMSO) δ: 11.79 (s, 1H), 7.66 (d, *J* = 7.5 Hz, 2H), 7.43 (t, *J* = 7.5 Hz, 2H), 7.37 (t, *J* = 7.3 Hz, 1H), 6.82 (d, *J* = 1.8 Hz, 1H). ^{13}C{^{1}H} NMR (101 MHz, d_6-DMSO) δ: 173.4, 134.3, 130.1, 129.3, 129.0, 125.3, 98.6.

4-(3-nitrophenyl)thiazol-2-one (11)

^{1}H NMR (400 MHz, d_6-DMSO) δ: 12.08 (s, 1H), 8.54 (t, *J* = 2.0 Hz, 1H), 8.22-8.18 (m, 1H), 8.12 (ddd, *J* = 7.9, 1.9, 1.0 Hz, 1H), 7.73 (t, *J* = 8.0 Hz, 1H), 7.17 (d, *J* = 1.5 Hz, 1H). ^{13}C{^{1}H} NMR (101 MHz, d_6-DMSO) δ: 173.1, 172.5, 148.7, 132.2, 131.5, 130.9, 123.4, 119.9, 101.9.

4-(4-chlorophenyl)thiazol-2-one (12)

^{1}H NMR (400 MHz, d_6-DMSO) δ: 11.84 (s, 1H), 7.73-7.64 (m, 2H), 7.55-7.46 (m, 2H), 6.89 (d, *J* = 1.8 Hz, 1H). ^{13}C{^{1}H} NMR (101 MHz, d_6-DMSO) δ: 172.8, 133.0, 132.7, 128.9, 128.5, 126.6, 99.1.

4-(3,4-dichlorophenyl)thiazol-2-one (13)

^{1}H NMR (400 MHz, d_6-DMSO) δ: 11.86 (s, 2H), 7.96 (d, *J* = 2.1 Hz, 2H), 7.73-7.61 (m, 4H), 7.04 (d, *J* = 1.9 Hz, 2H). ^{13}C{^{1}H} NMR (101 MHz, d_6-DMSO) δ: 172.6, 131.7, 131.5, 131.0, 130.8, 130.0, 126.6, 124.9, 100.8.

4-(2-oxo-2,3-dihydrothiazol-4-yl)benzonitrile (14)

^{1}H NMR (400 MHz, d_6-DMSO) δ: 11.99 (s, 1H), 7.94 - 7.90 (m, 2H), 7.88-7.83 (m, 2H), 7.17 (d, *J* = 1.9 Hz, 1H). ^{13}C{^{1}H} (101 MHz, d_6-DMSO) δ: 173.1, 134.0, 133.3, 128.4, 125.9, 119.0, 111.0, 102.9.

4-(4-methoxyphenyl)thiazol-2-one (15)

^{1}H NMR (400 MHz, d_6-DMSO) δ: 11.69 (s, 1H), 7.63-7.56 (m, 2H), 7.02-6.94 (m, 2H), 6.63 (d, *J* = 2.1 Hz, 2H), 3.78 (s, 3H). ^{13}C{^{1}H} NMR (101 MHz, d_6-DMSO) δ: 173.5, 159.8, 134.1, 126.8, 122.8, 114.7, 96.3, 55.7.

General considerations: carbon-11 radiolabelling procedures

Starting materials were purchased from either Sigma-Aldrich or VWR International and used without further purification. Quality control analysis of radioactive product mixtures was performed by high performance liquid chromatography (HPLC) using an Agilent 1100 system with in-line radioactivity and diode array detectors (UV) fitted with an Agilent Eclipse XDB -C_{18} column (5 µm, 4.6 x 150 mm) and an eluent mixture of acetonitrile and ammonium formate at a flow of 1.5 mL/min. Radioactivity measurements were performed using a Isomed 2000 dose calibrator. A co-injection with authentic unlabelled reference standard was used to confirm the presence of the product as evidenced by overlapping UV and radioactivity peaks.

[^{11}C]Carbon disulfide production

[^{11}C]carbon dioxide was produced by the nuclear $^{14}N(p,\alpha)^{11}C$ reaction using a Siemens Eclipse HP cyclotron by 11 MeV proton bombardment of a gas target charged with nitrogen gas doped with 1% oxygen. The typical irradiation conditions used for these experiments were: 15 µA, 5 minute beam. [^{11}C]CO_2 was converted to [^{11}C]CH_3I using the commercially-available Synthra gas phase methyl iodide module. [^{11}C]CH_3I was then delivered under a flow of helium gas (~10 sscm) into a preheated reactor column (Omnifit, 10 mm x 150 mm, part no. 56003) packed with a mixture of elemental sulfur (1.0 g) and sand (2.0 g) and plugged at either end with quartz wool. The reactor column was preheated to 450°C using a Carbolite horizontal single zone tube furnace (model MTF10/25/130) for several minutes prior to delivery of the [^{11}C]CH_3I gas stream. The resultant [^{11}C]CS_2-helium gas stream was delivered to a 20 mL glass reaction vial, containing a trapping solvent and fitted with a rubber septum cap. A bag was attached to the vial exhaust in order to contain any radioactive gases that were not trapped in solution. The flow of radioactivity was monitored by placing pin-diode radioactivity detectors at column oven and the reaction vial. Once the radioactivity had peaked in the trapping vial the gas flow was stopped and vial inlet and outlet needles removed.

General carbon-11 thiocyanation procedure

In a typical labelling procedure, the [^{11}C]CS_2 gas stream was bubbled through acetonitrile (1 mL) in a 5 mL V-shaped glass reaction vial. Once the radioactivity of the solution reached a maximum, the flow was stopped, inlet and outlet needles removed and methanolic ammonia solution (50 µL, 2 M, 0.1 mmol) added. The reaction mixture was then heated at 90°C for 5 minutes to generate ammonium [^{11}C]thiocyanate. After this time, the vial was removed from the heater and a solution of bromoacetophenone precursor (5 mg) in acetonitrile (0.1 mL) added. The reaction mixture was stirred for a further 5-10 minutes at room temperature after which the radioactivity was measured and an

aliquot of the reaction solution removed and analysed by analytical radio-HPLC. Radiochemical yields (non-isolated) determined from the peak areas of the analytical radioHPLC traces of the crude product based on conversion from [^{11}C]CH_3I. Representative analytical radio-HPLC traces of labelling reactions and UV traces of reference compounds are shown below.

General carbon-11 thiazolone synthesis

To a solution of carbon-11 labelled phenacyl thiocyanate, prepared via the general carbon-11 thiocyanation procedure outlined above, was added H_2SO_4 (50 % v/v, 0.5 mL) and glacial acetic acid (0.5 mL). The reaction mixture heated to 90 ^{o}C for 5 minutes and an aliquot of the reaction solution removed and analysed by analytical radio-HPLC.

Analytical HPLC Data

Crude radio-HPLC trace of labelling reaction (top), UV-HPLC trace spiked with the unlabelled reference compound (middle) and crude UV-HPLC trace of labelling reaction (bottom).

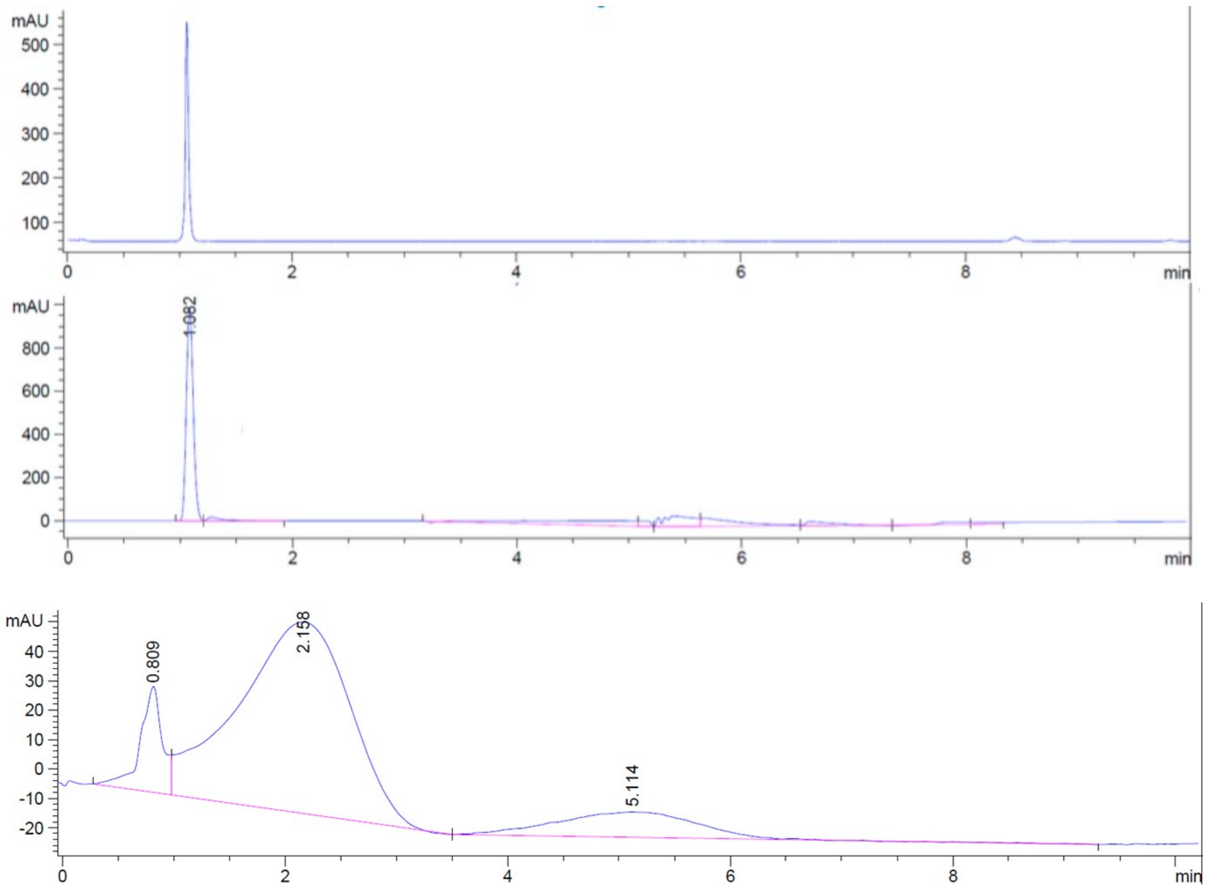

ammonium [^{11}C]thiocyanate

benzyl [11C]thiocyanate (1)

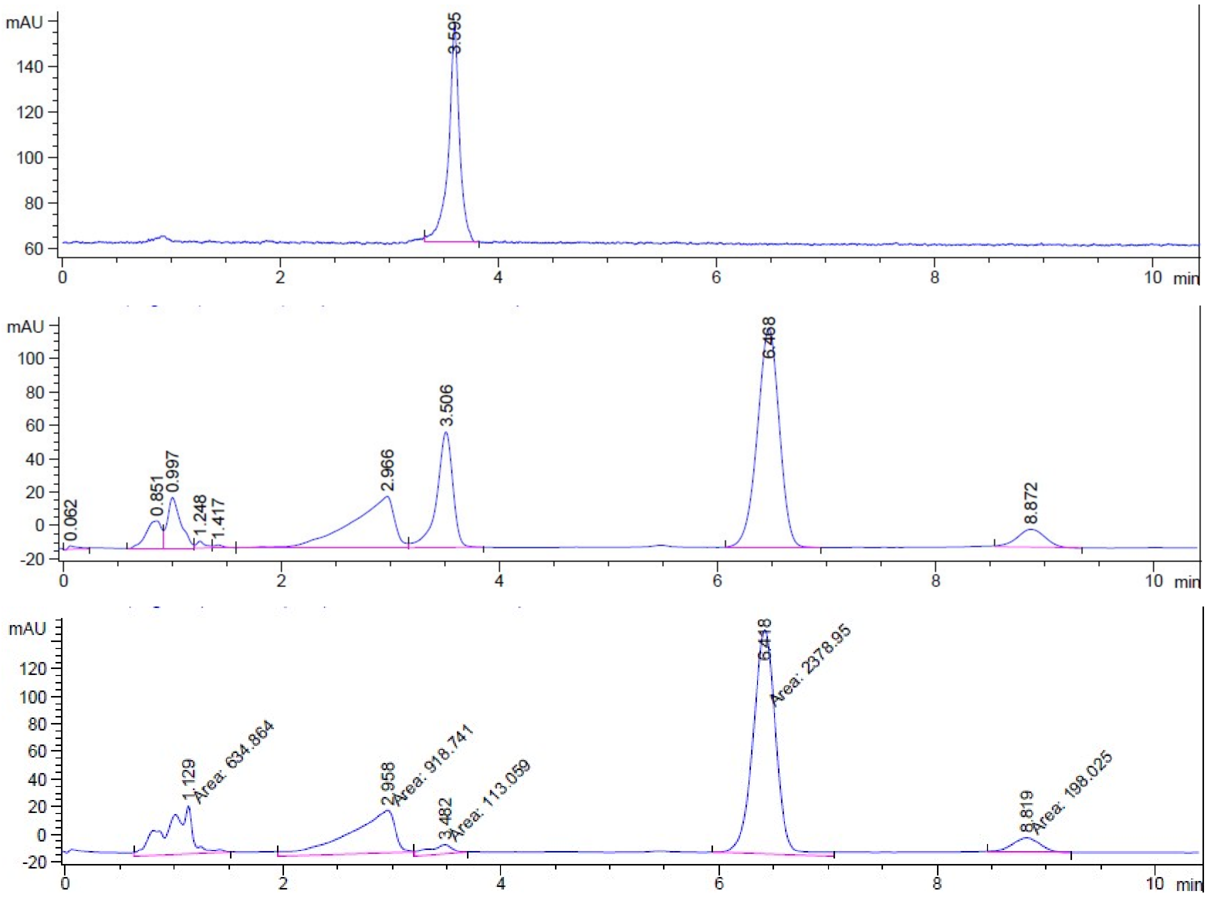

1-phenyl-2-[11C]thiocyanatoethanone (2)

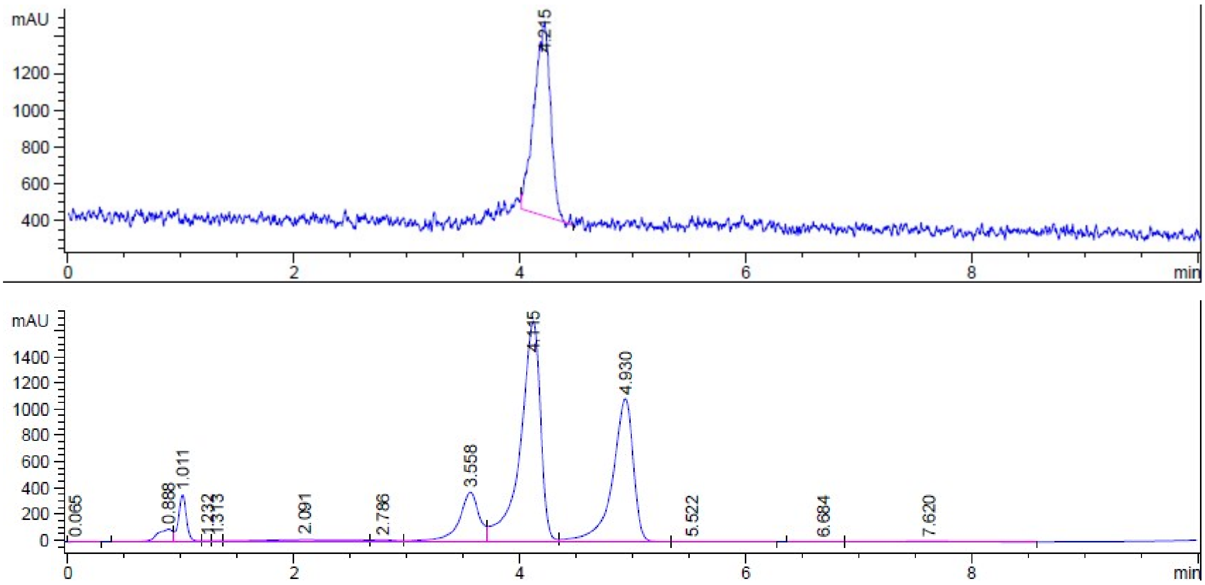

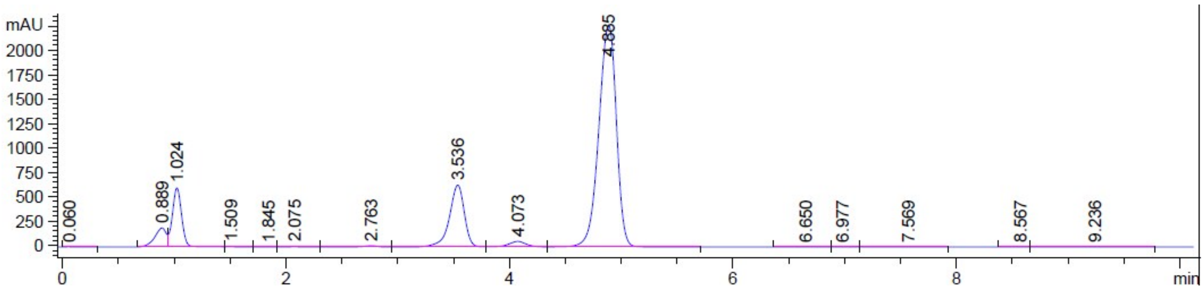

1-(3-nitrophenyl)-2-[^{11}C]thiocyanatoethanone (3)

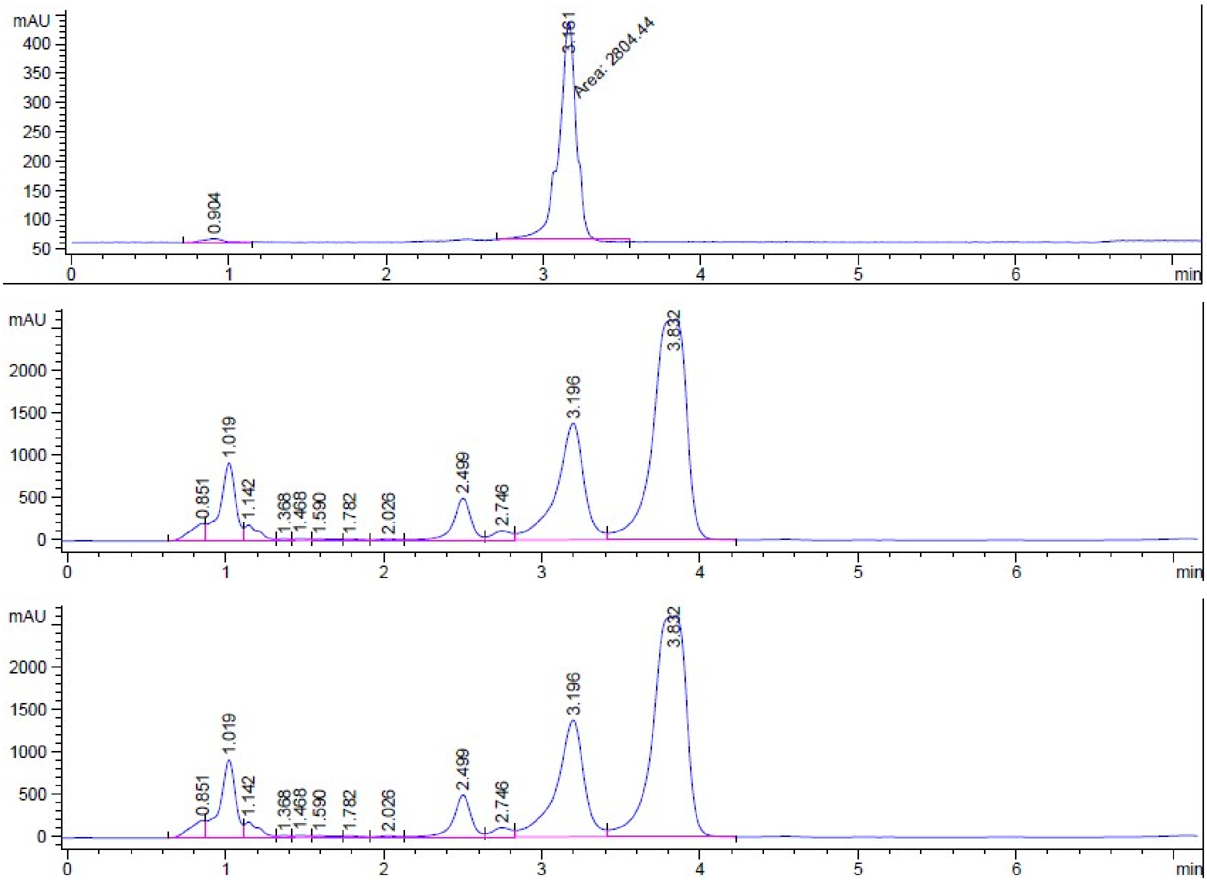

1-(4-chlorophenyl)-2-[^{11}C]thiocyanatoethanone (4)

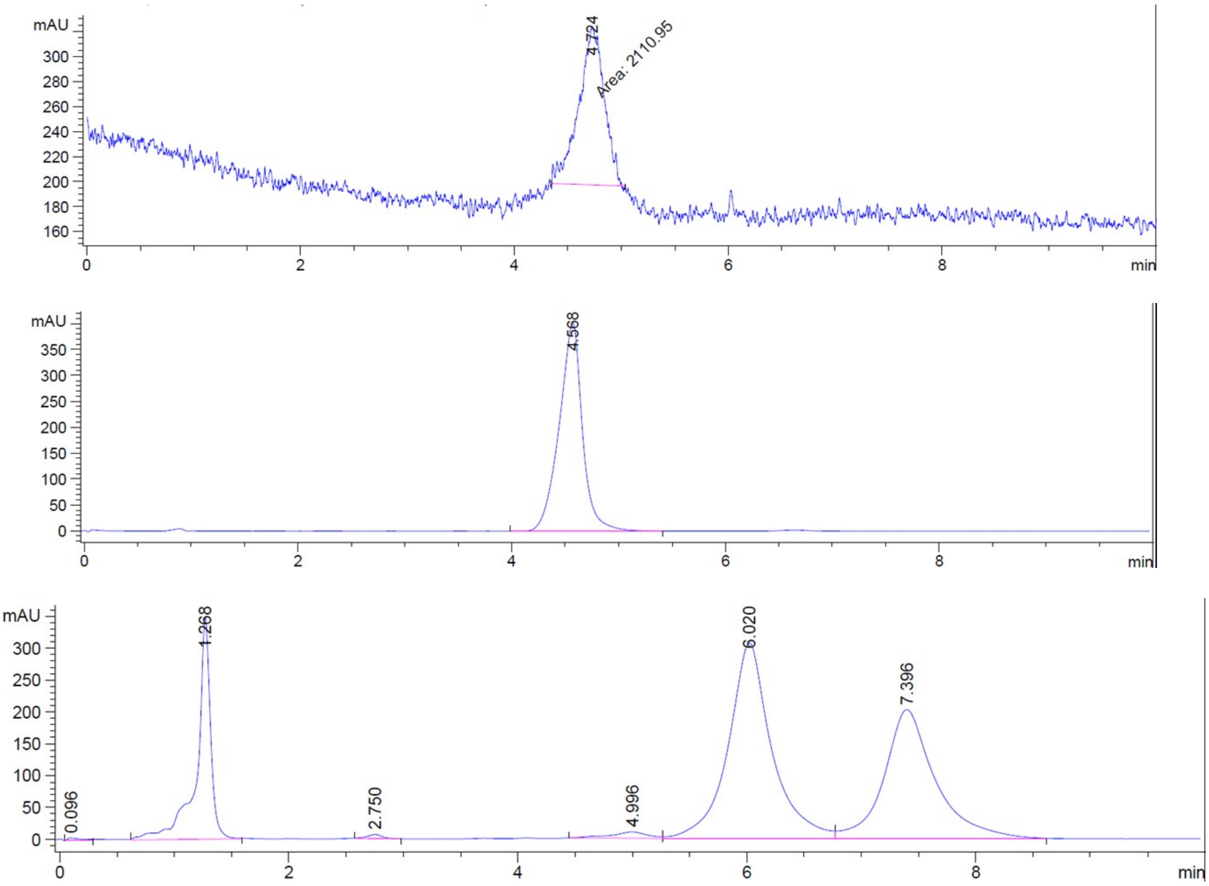

1-(3,4-dichlorophenyl)-2-[^{11}C]thiocyanatoethanone (5)

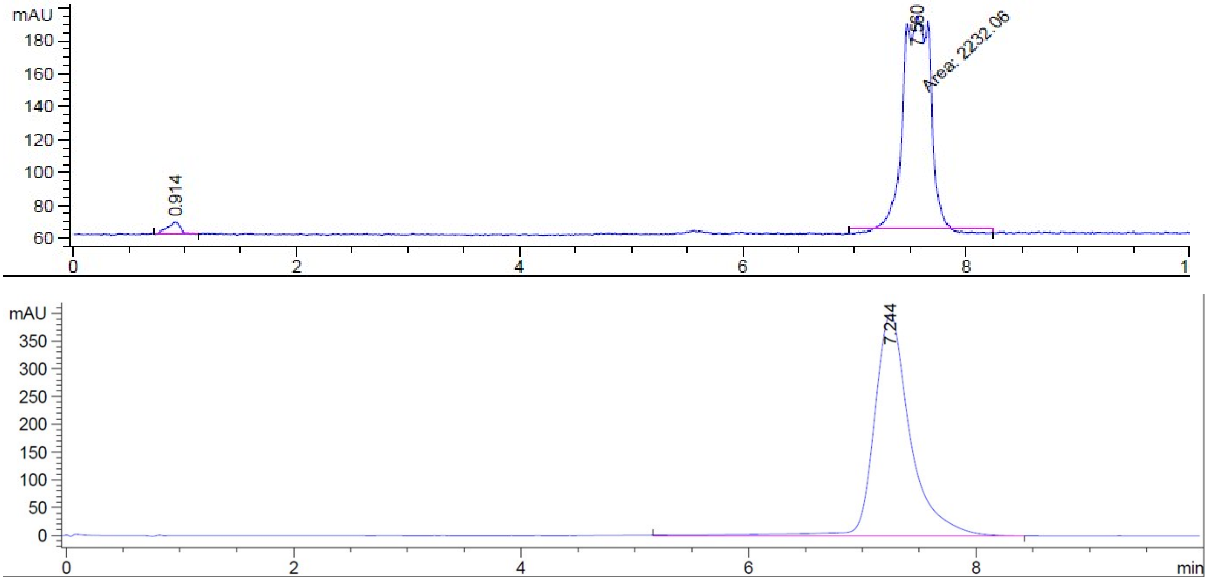

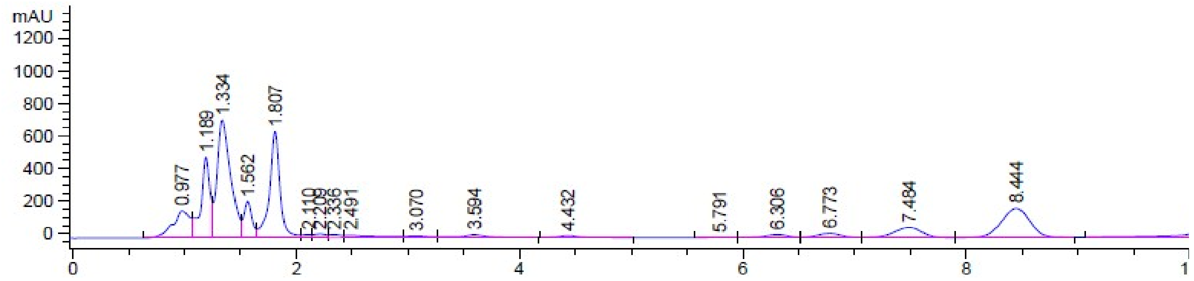

1-(4-benzonitrile)-2-[^{11}C]thiocyanatoethanone (6)

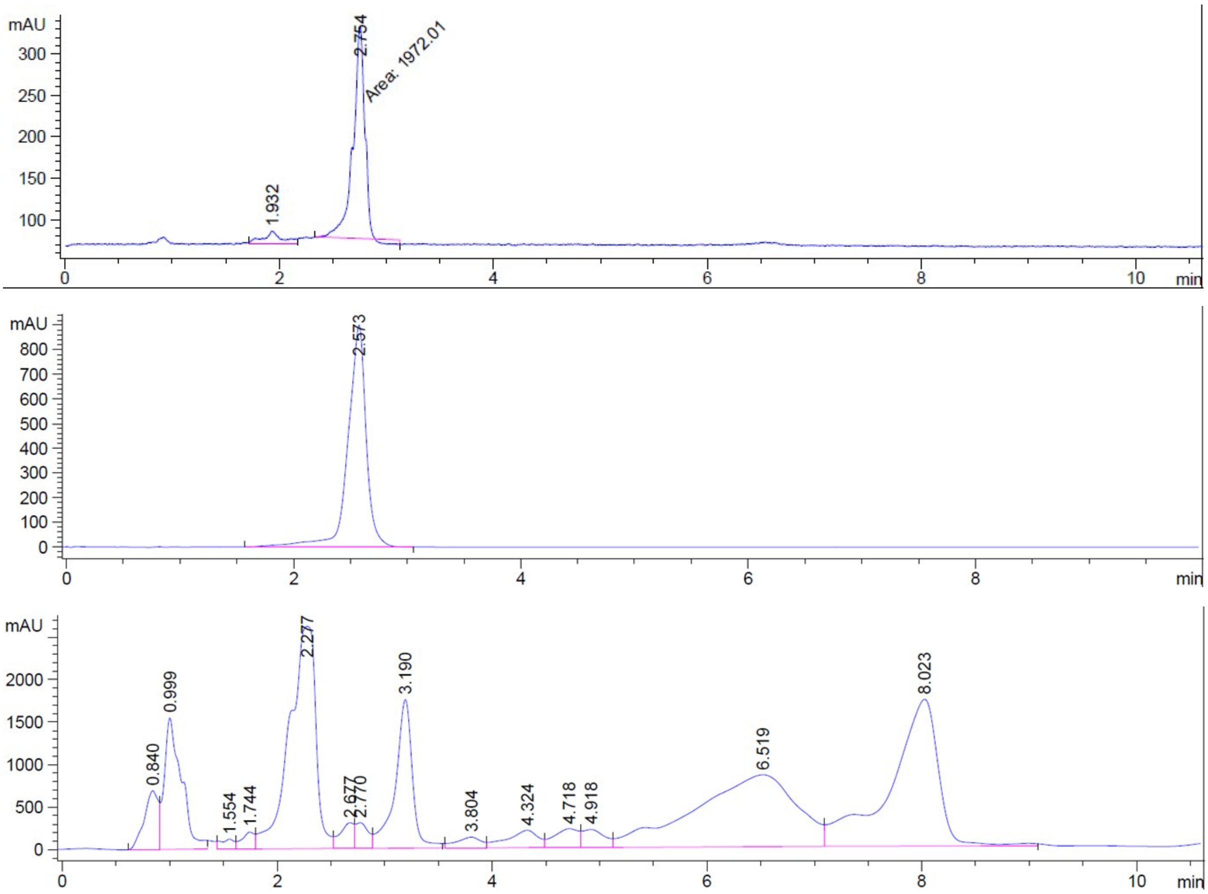

1-(4-methoxyphenyl)-2-[^{11}C]thiocyanatoethanone (7)

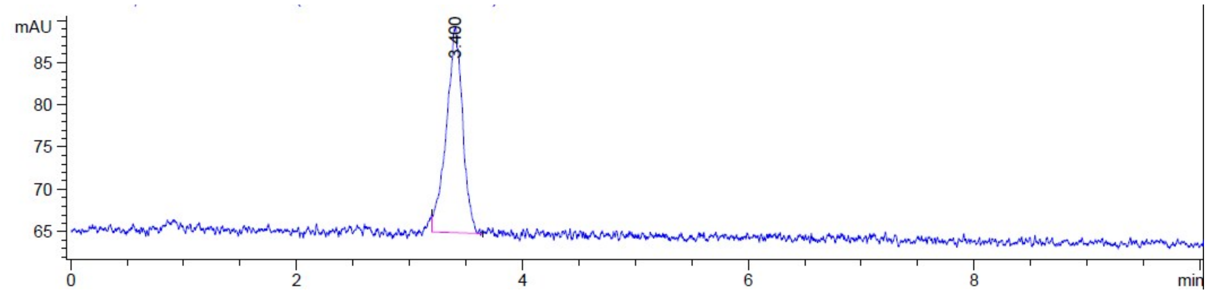

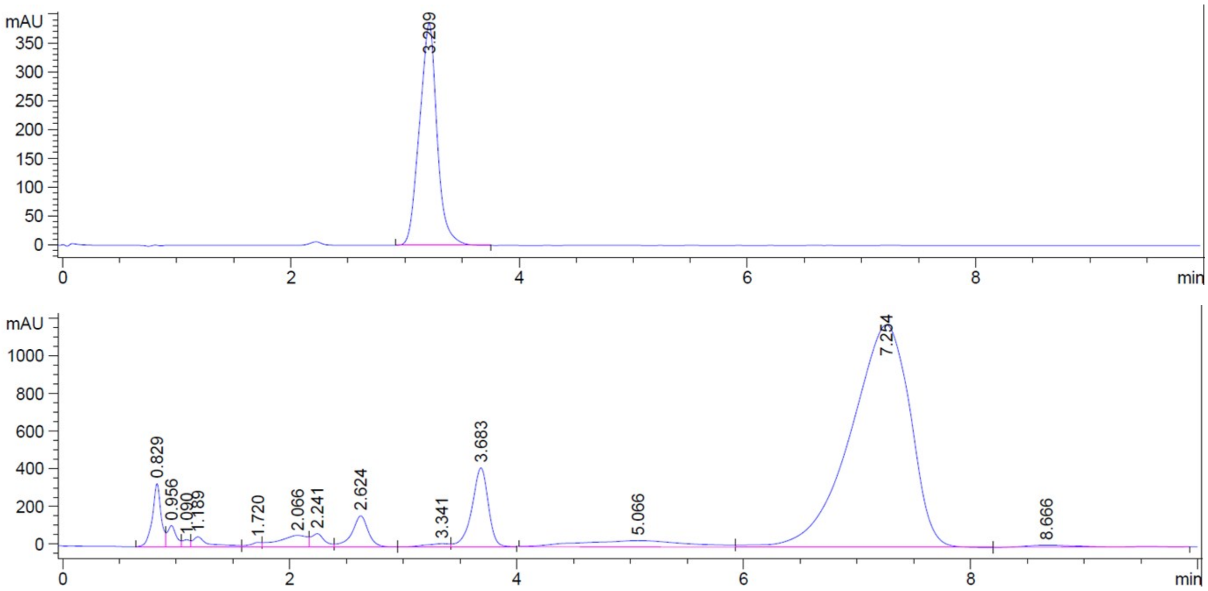

1,2-diphenyl-2-[^{11}C]thiocyanatoethanone (8)

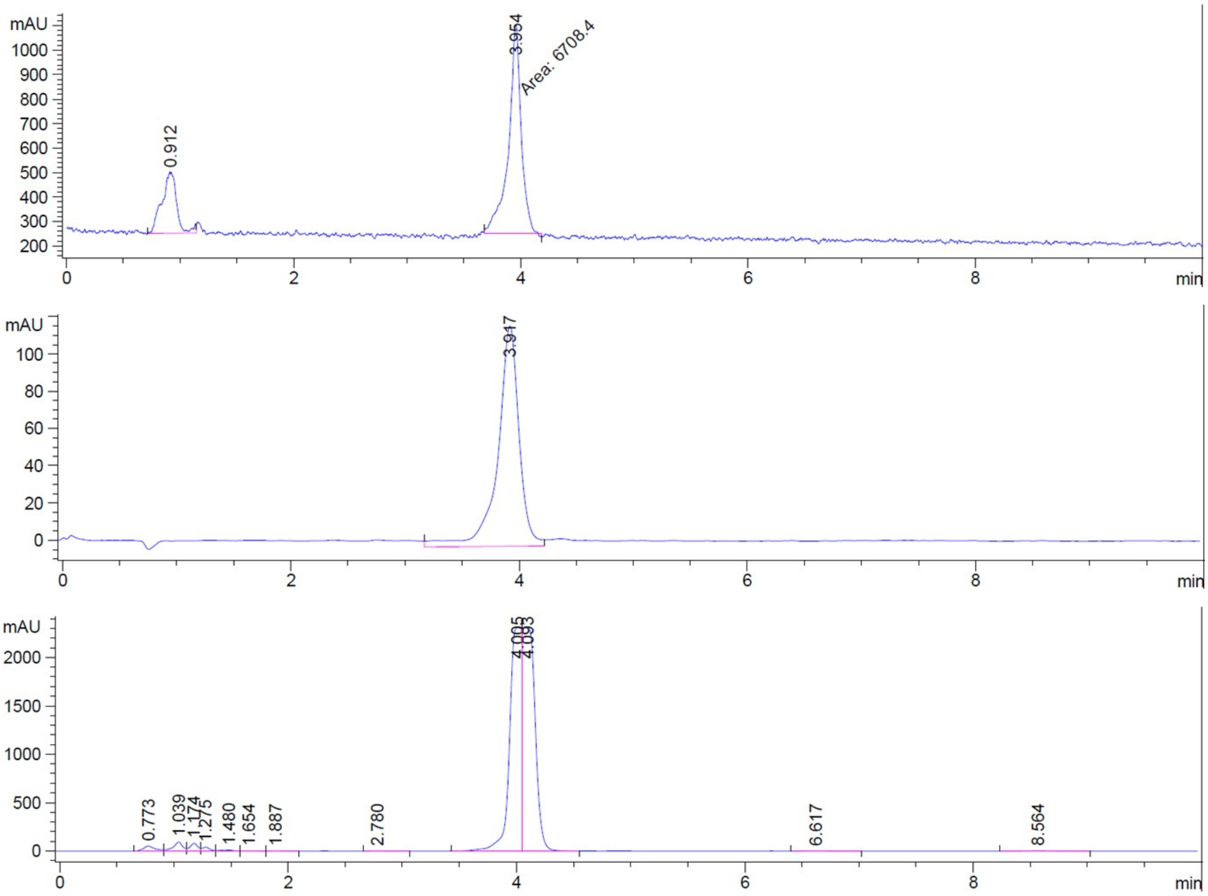

1,3,4,6-tetra-*O*-acetyl-2-[^{11}C]thiocyanato-D-mannopyranose (9)

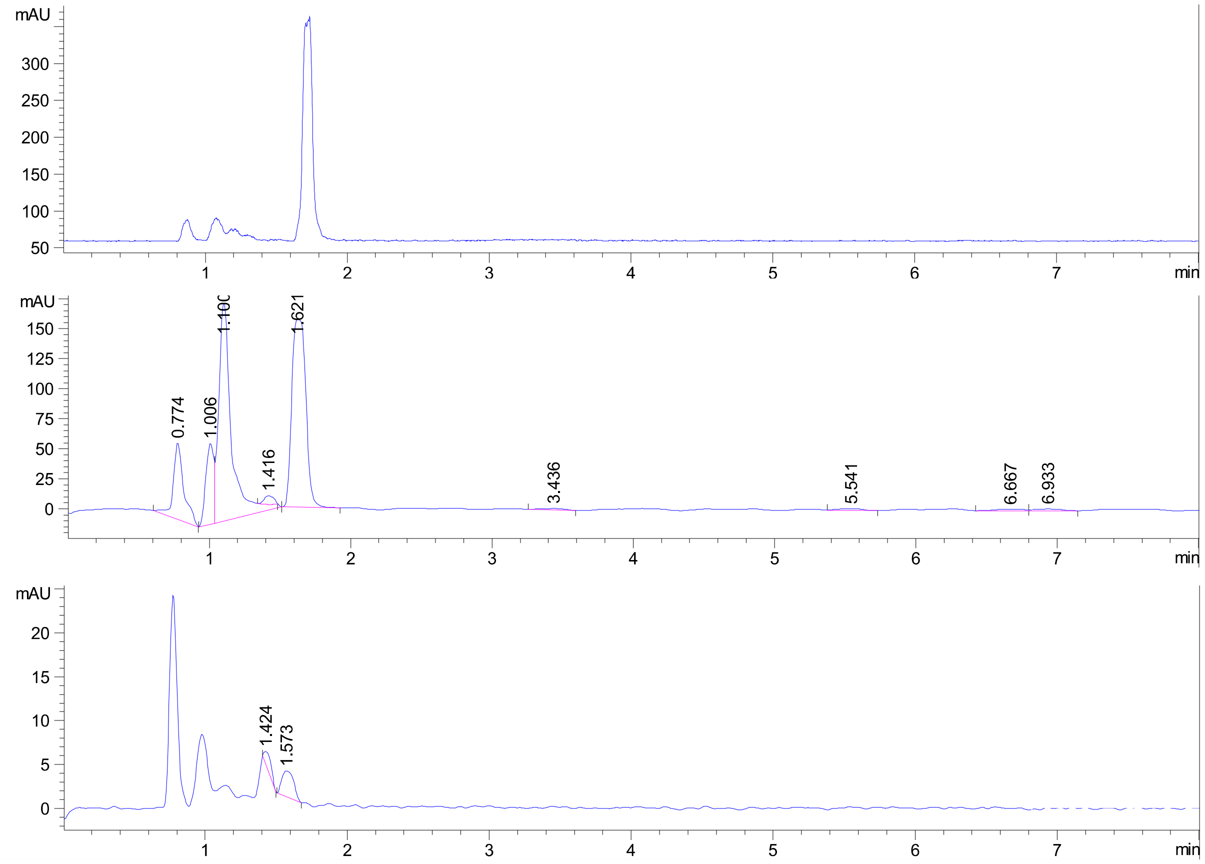

4-phenylthiazol-2-[^{11}C]one (10)

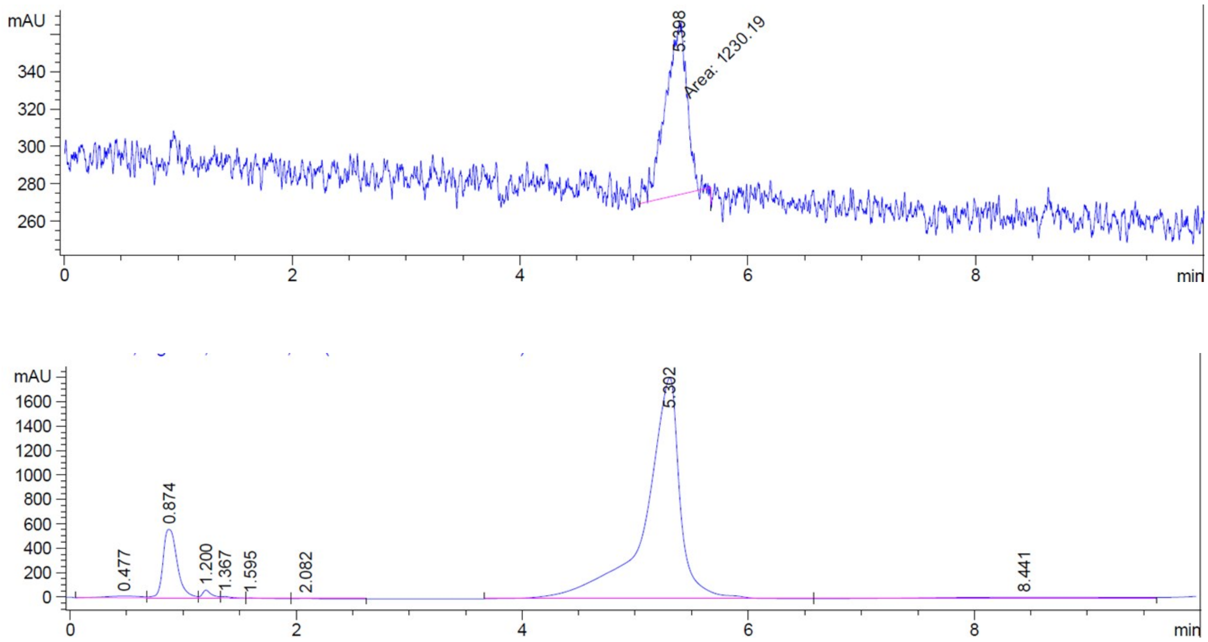

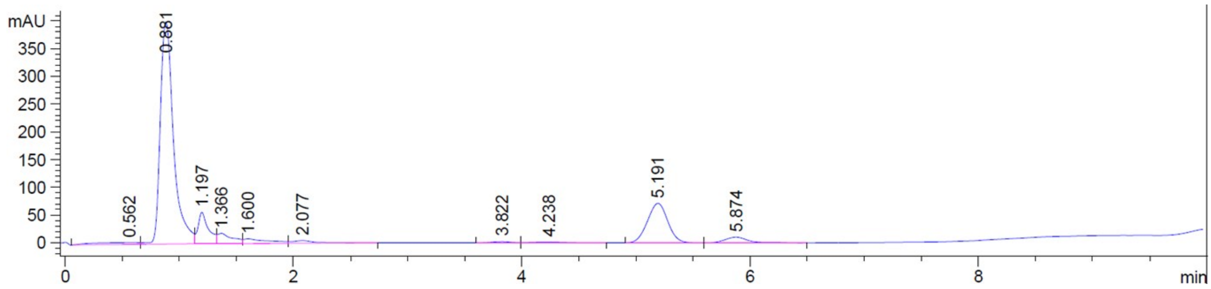

4-(3-nitrophenyl)thiazol-2-[^{11}C]one (11)

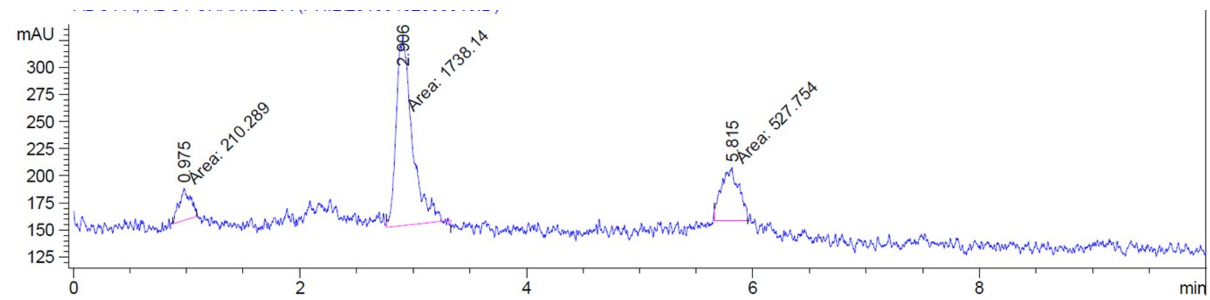

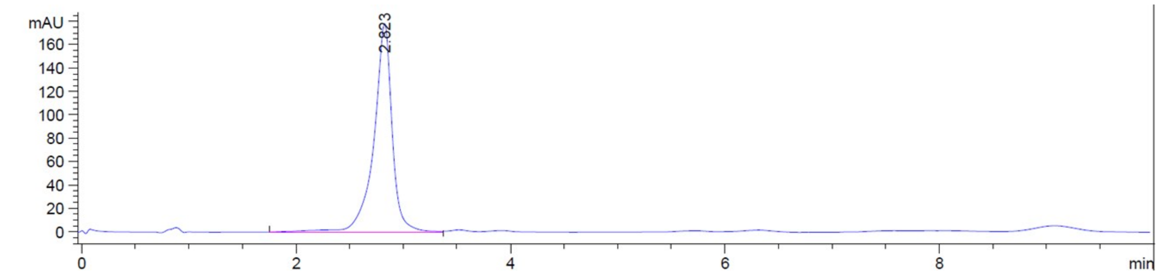

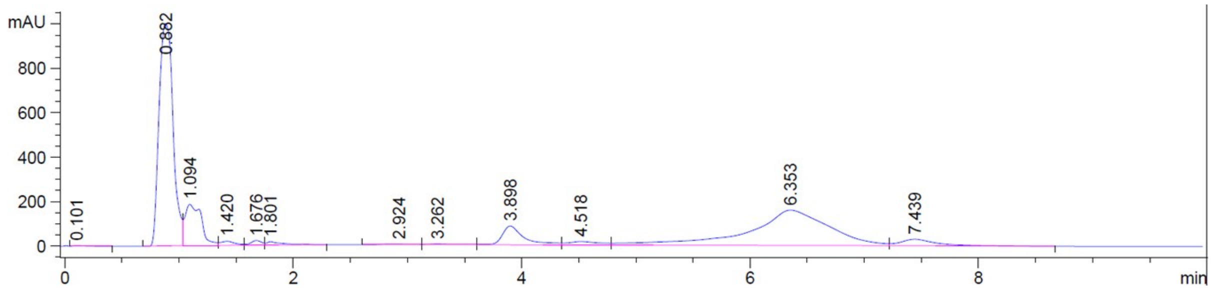

4-(4-chlorophenyl)thiazol-2-[^{11}C]one (12)

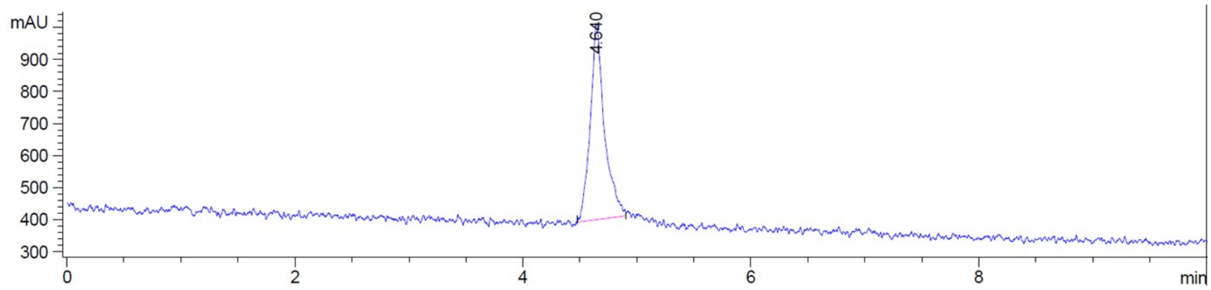

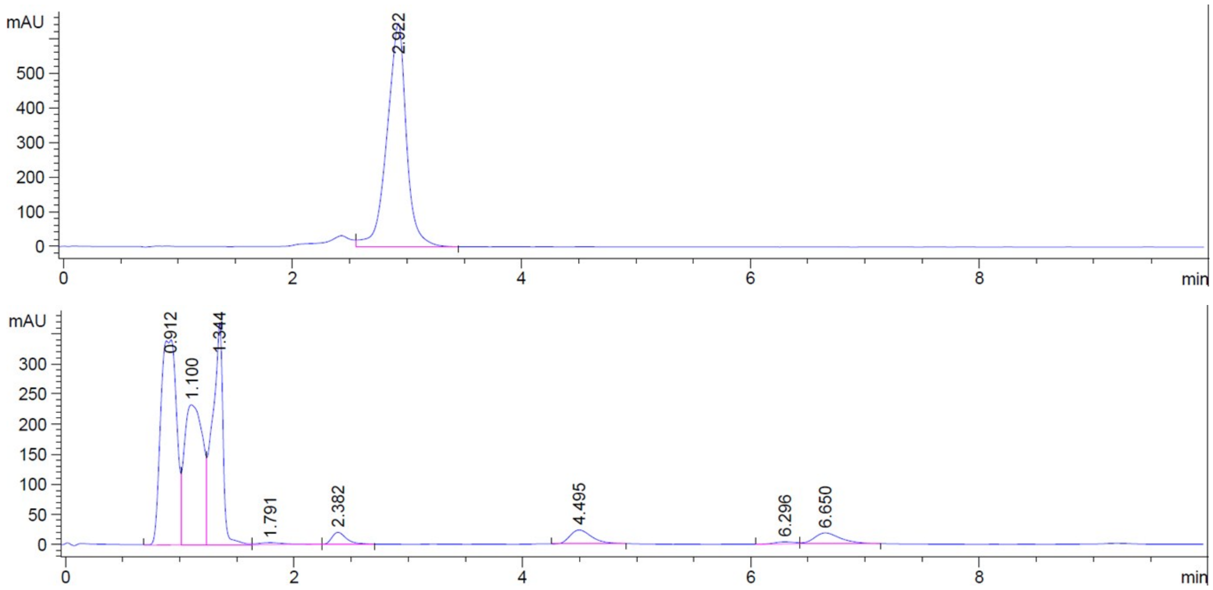

[^{11}C]4-(3,4-dichlorophenyl)thiazol-2-one (13)

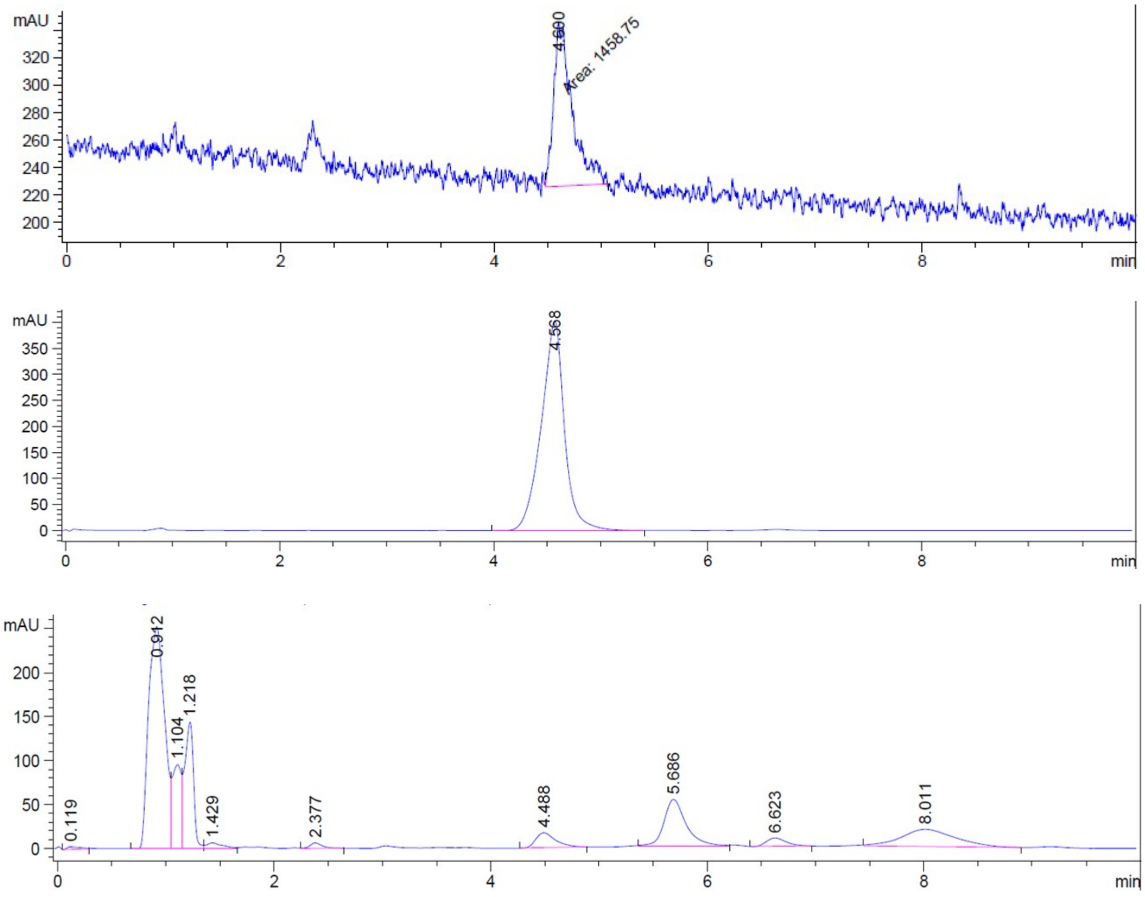

4-(2-oxo-2,3-dihydro[^{11}C]thiazol-4-yl)benzonitrile (14)

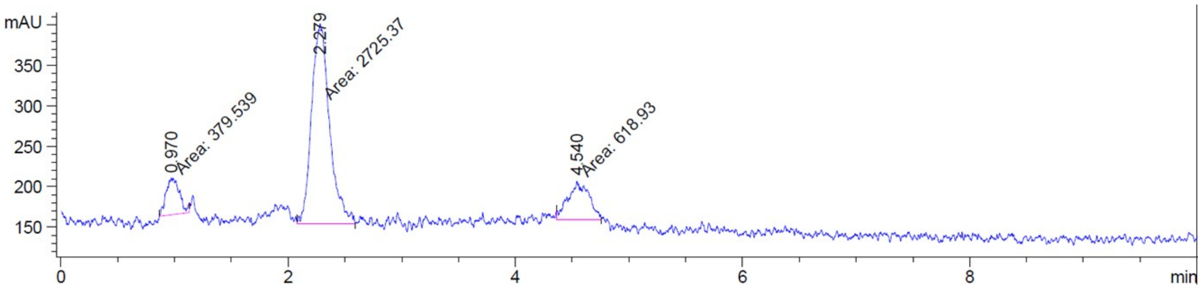

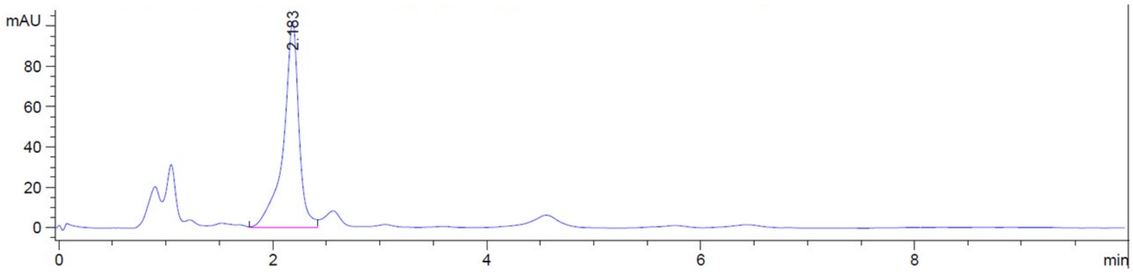

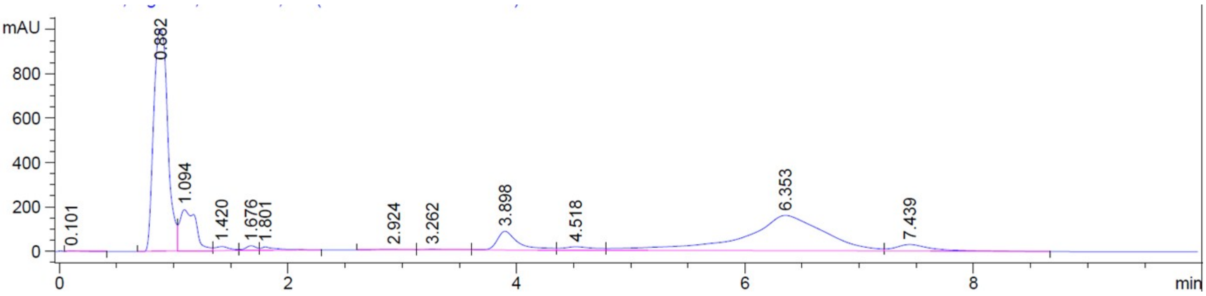

4-(4-methoxyphenyl)thiazol-2-[^{11}C]one (15)

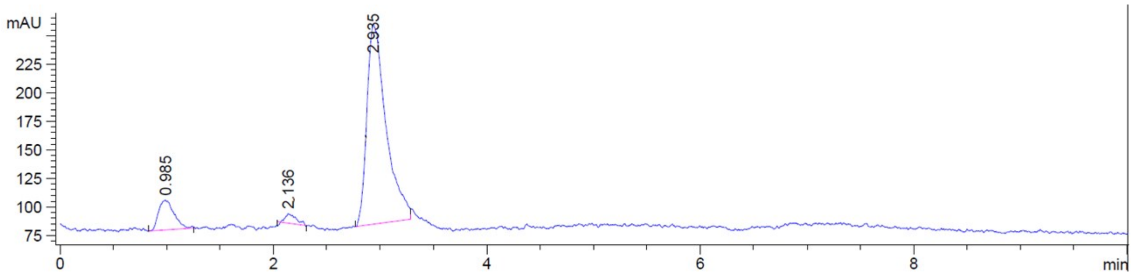

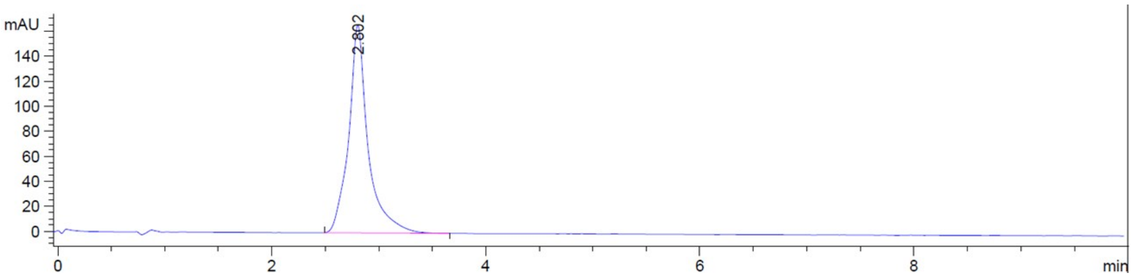

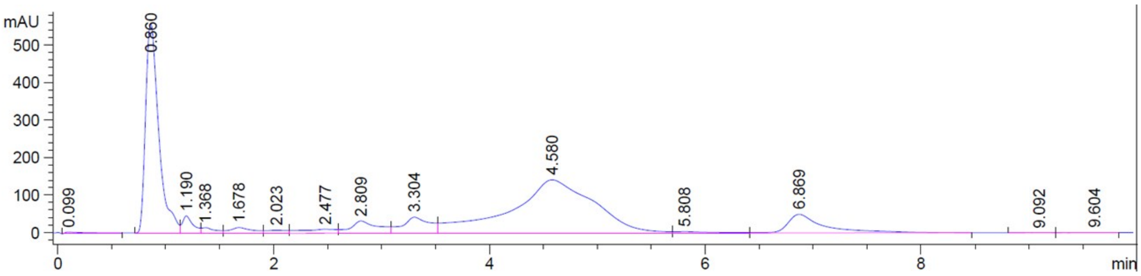

References

[1] L. Zhao, D. Cao, T. Chen, Y. Wang, Z. Miao, Y. Xu, W. Chen, X. Wang, Y. Li, Z. Du, B. Xiong, J. Li, C. Xu, N. Zhang, J. He and J. Shen, J. Med. Chem., 2013, 56, 3833–3851.

[2] K. Pihlaja, V. Ovcharenko, E. Kolehmainen, K. Laihia, W. M. F. Fabian, H. Dehne, A. Perjéssy, M. Kleist, J. Teller and Z. Šusteková, J. Chem. Soc. Perkin Trans. 2, 2002, 2, 329–336.
